# Supplementary material for: Synaptic FUS accumulation triggers early misregulation of synaptic RNAs in a mouse model of ALS
Source: Nat Commun. 2021 May 21;12:3027. doi: 10.1038/s41467-021-23188-8 (PMC8140117; doi:10.1038/s41467-021-23188-8)
Supplement: Supplementary file 1 — Supplementary Information [file 41467_2021_23188_MOESM1_ESM.pdf]

Supplementary Information

Synaptic FUS accumulation triggers early misregulation of synaptic RNAs in a mouse model of ALS

Sonu Sahadevan[1*], Katharina M. Hembach[1,2*], Elena Tantardini[1], Manuela Pérez-Berlanga[1], Marian Hruska-Plochan[1], Salim Megat[3], Julien Weber[1], Petra Schwarz[4], Luc Dupuis[3], Mark D. Robinson[2], Pierre De Rossi[1], Magdalini Polymenidou[1,#]

[1]Department of Quantitative Biomedicine, University of Zurich
[2]Department of Molecular Life Sciences and SIB Swiss Institute of Bioinformatics, University of Zurich
[3]Inserm, University of Strasbourg
[4]Institute of Neuropathology, University Hospital Zurich

*These authors contributed equally to this work

#Author for correspondence: magdalini.polymenidou@uzh.ch

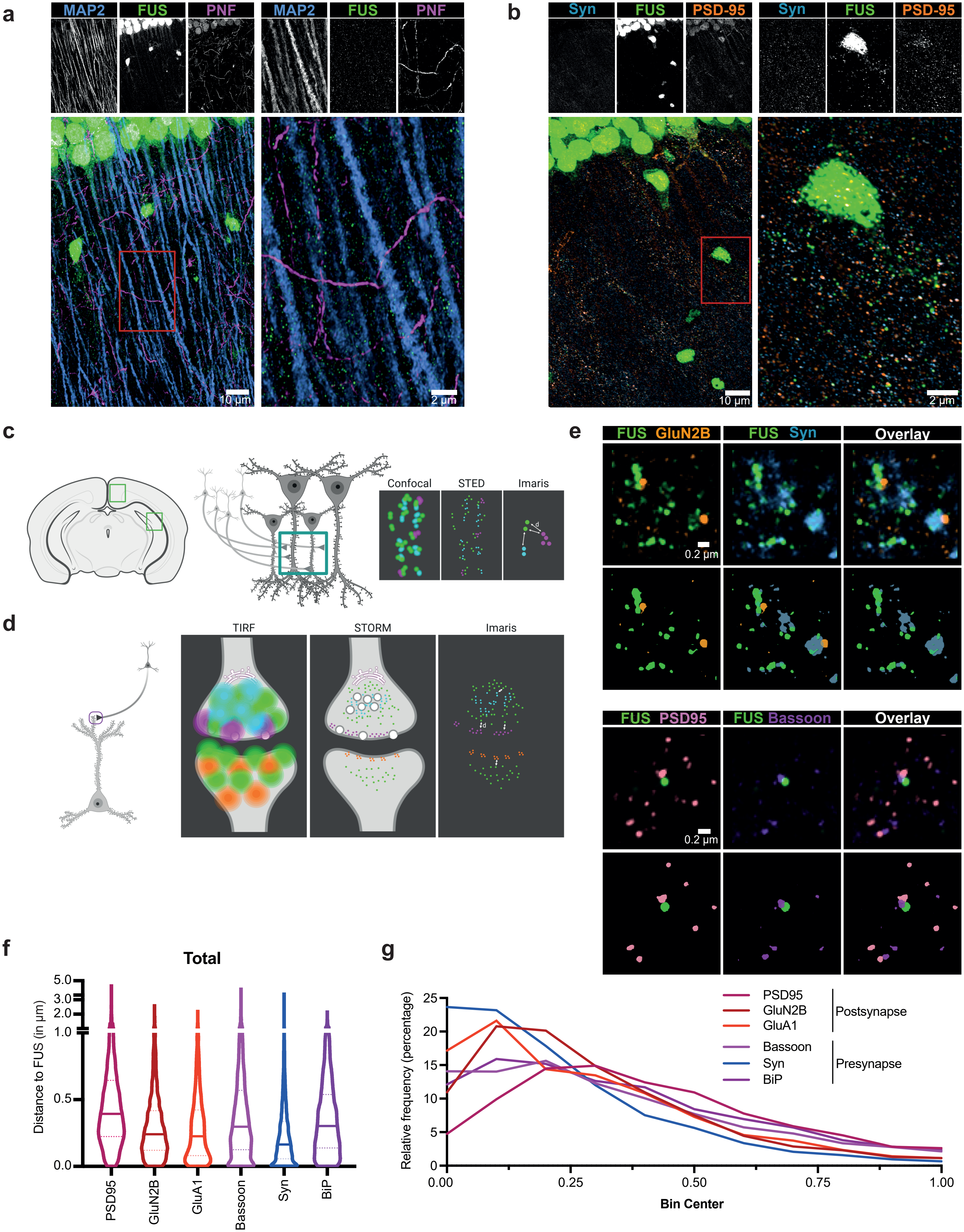

Supplementary Fig. 1 FUS is enriched at the presynaptic compartment

Supplementary Fig. 1 FUS is enriched at the presynaptic compartment

(**a**) Confocal images showing the distribution of Fused in sarcoma (FUS) (green) in the molecular layer of the CA1 hippocampal area along with microtubule-associated protein 2 (MAP2) (blue) and phospho-neurofilament (PNF)(magenta). Left panel shows the overview and the right panel, the zoomed-in area labelled with the red box on the left panel. n= 5, independent mouse (**b**) Similar confocal images showing FUS (green) along with postsynaptic density protein 95 (PSD95) (orange) and Synapsin 1 (Syn, blue). n= 5, independent mouse. (**c**) Schematic of the workflow for distance calculation after STED imaging. (**d**) Schematic of the workflow for distance calculation after STORM imaging. Colors and structures depicted in this panel do not represent specific labels or structures and are only shown for illustration purposes. (**e**) Representative images of STORM imaging for FUS-GluN2B-Synapsin 1 and FUS-PSD95-Bassoon. n=3 independent neuronal culture, with n=17 fields of view analyzed for postsynaptic markers and 31 fields of view analyzed for presynaptic markers (**f**) Violin graph representing the distance distribution between FUS and synaptic markers. (**g**) Binning distribution showing the distance between FUS and the markers (in relative frequency) for PSD95, GluN2B, GluA1, Bassoon, Synapsin and BiP (endoplasmic reticulum marker).

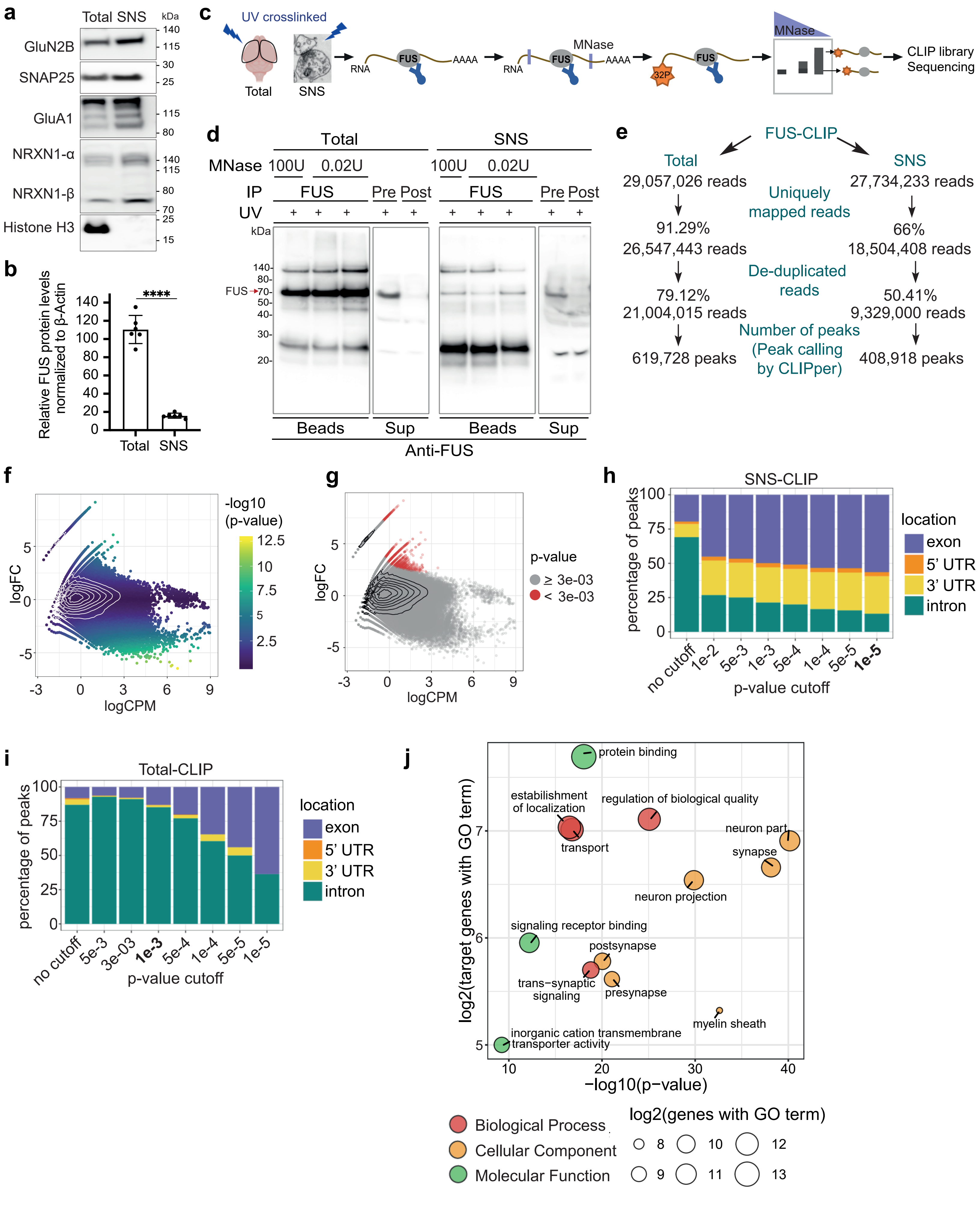

Supplementary Fig. 2 CLIP-seq on cortical synaptoneurosomes identified FUS-associated pre- and postsynaptic RNAs

Supplementary Fig. 2 CLIP-seq on cortical synaptoneurosomes identified FUS-associated pre- and postsynaptic RNAs

(**a**) Western blot of synaptic proteins GluN2B (NMDAR subunit), Synaptosomal-associated protein 25 (SNAP25), GluA1 (AMPAR subunit), Neurexin 1 (NRXN1), nuclear protein (Histone H3) in total cortex and synaptoneurosomes (SNS). Source data are provided as a Source Data file. (**b**) Quantification of Fused in sarcoma (FUS) in SNS compared to the total cortex samples extrapolated from western blot (Fig. 2b). The bar graph represent mean ± SD. The SNS and corresponding total samples were prepared from cortices derived from six different mice (n=6). ****p<0.0001, two-tailed, unpaired t-test (**c**) Schematic of CLIP-seq workflow from total cortex and SNS from mouse cortex. (**d**) Immunoblot showing efficient immunoprecipitation of FUS from total cortex and synaptoneurosomes. n=3 independent experiments (**e**) Flow chart illustrating the reads analyzed to define FUS peaks in total and synaptoneurosomes. (**f**) MA-plot of CLIPper peaks predicted in the total cortex CLIP-seq sample. logCPM is the average log2CPM of each peak in the total cortex and synaptoneurosome sample and logFC is the log2 fold-change between the number of reads in the total cortex and synaptoneurosome sample. (**g**) Same MA-plot as (f) showing the selected, total cortex-specific peaks (p-value cutoff of 3e-03) in red. (**h**) Bar plot of different sets of synaptoneurosome peaks and their location in genes. The p-value cutoff of each set is on the x-axis and no cutoff refers to the full list of all predicted synaptoneurosome CLIPper peaks. The selected cutoff is in bold. (**i**) Bar plot of different sets of total cortex peaks and their location in genes. The p-value cutoff of each set is on the x-axis and no cutoff refers to the full list of all predicted synaptoneurosome CLIPper peaks. The selected cutoff is in bold. (**j**) GO terms enriched among the synapse-specific FUS RNA targets. One-sided hypergeometric test, p-value unadjusted for multiple comparisons. P-values shown in (f-i) are computed by likelihood-ration test and unadjusted for multiple comparisons.

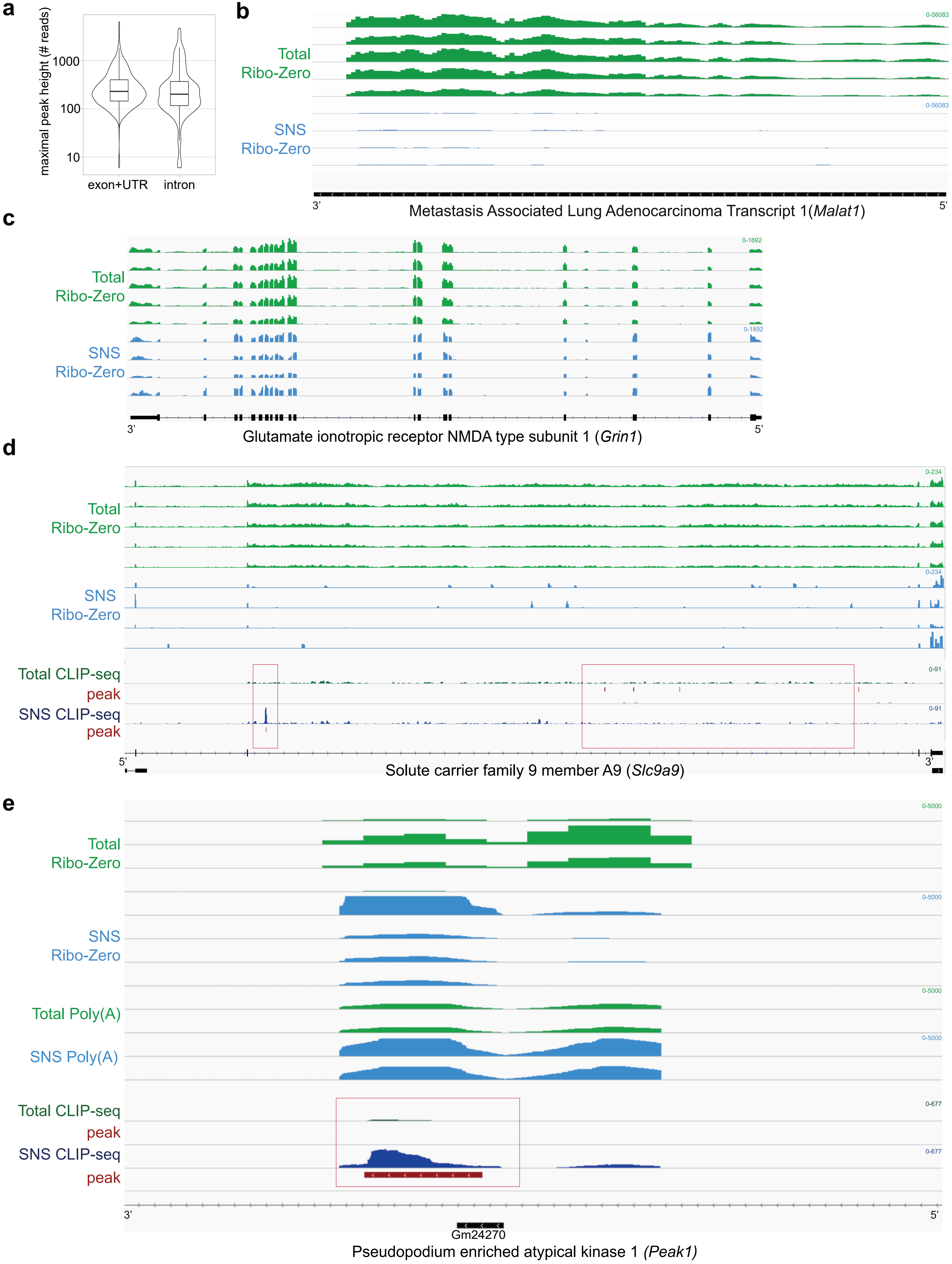

Supplementary Fig. 3 Intronic coverage and FUS peak locations in RNA targets of FUS

Supplementary Fig. 3 Intronic coverage and FUS peak locations in the RNA targets of FUS

(**a**) Maximal number of synaptoneurosome FUS-CLIP reads from exon and UTR (n = 1301 peaks), and introns (n = 227 peaks). Data is represented as violin and box plots that indicate median (middle line), 25th, 75th percentile (box) and the lower/upper whiskers extend to the minimal/maximal value no further than 1.5 × inter-quartile range from the hinge. (**b-c**) Read coverage of *Malat1* (**b**) and *Grin1* (**c**) in Ribo-zero RNA-seq data from synaptoneurosomes and total cortex. (**d-e**) Intronic read coverage from Ribo-zero RNA-seq data and FUS-CLIP peaks on *Slc9a9* (**d**) and (**e**) *Peak1* in total and SNS samples.

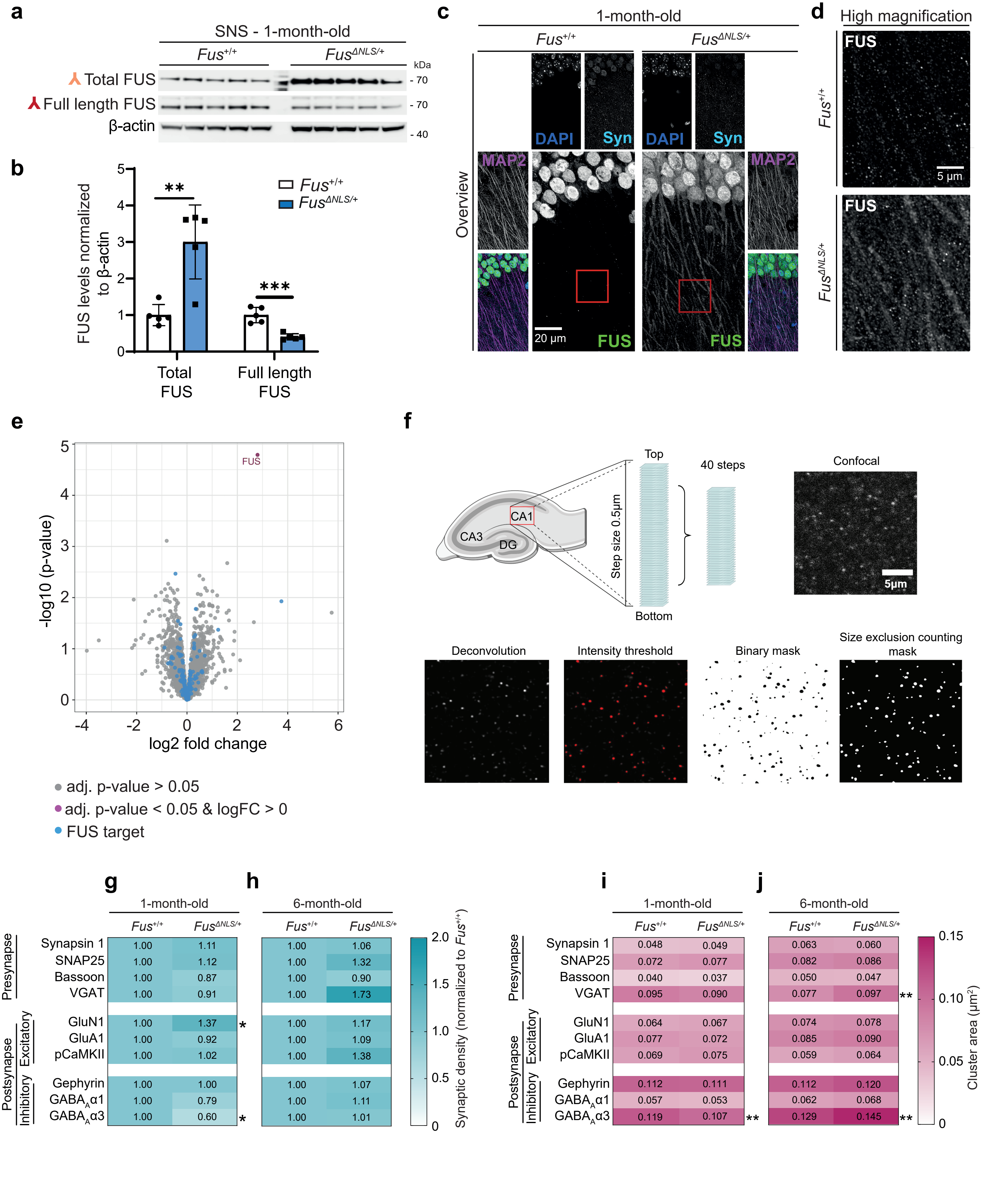

	g: 1-month-old Fus+/+	g: 1-month-old FusΔNLS/+	h: 6-month-old Fus+/+	h: 6-month-old FusΔNLS/+	i: 1-month-old Fus+/+	i: 1-month-old FusΔNLS/+	j: 6-month-old Fus+/+	j: 6-month-old FusΔNLS/+
Synapsin 1	1.00	1.11	1.00	1.06	0.048	0.049	0.063	0.060
SNAP25	1.00	1.12	1.00	1.32	0.072	0.077	0.082	0.086
Bassoon	1.00	0.87	1.00	0.90	0.040	0.037	0.050	0.047
VGAT	1.00	0.91	1.00	1.73	0.095	0.090	0.077	0.097 **
GluN1	1.00	1.37 *	1.00	1.17	0.064	0.067	0.074	0.078
GluA1	1.00	0.92	1.00	1.09	0.077	0.072	0.085	0.090
pCaMKII	1.00	1.02	1.00	1.38	0.069	0.075	0.059	0.064
Gephyrin	1.00	1.00	1.00	1.07	0.112	0.111	0.112	0.120
$GABA_A\alpha1$	1.00	0.79	1.00	1.11	0.057	0.053	0.062	0.068
$GABA_A\alpha3$	1.00	0.60 *	1.00	1.01	0.119	0.107 **	0.129	0.145 **

Supplementary Fig. 4 Increased synaptic FUS localization in *Fus*$^{\Delta NLS/+}$ mice affect GABAergic synapses

Supplementary Fig. 4 Increased synaptic FUS localization in $Fus^{\Delta NLS/+}$ mice affect GABAergic synapses

(a) Western blot of total FUS, full length FUS and actin in synaptoneurosomes isolated from 1-month-old $Fus^{+/+}$ and $Fus^{\Delta NLS/+}$ mice. Source data are provided as a Source Data file. (**b**) Quantification of total FUS (p-value = 0.0028) and full length FUS levels (p-value = 0.0004) in synaptoneurosomes from $Fus^{+/+}$ and $Fus^{\Delta NLS/+}$ at 1 month of age. Error bars represent mean ± SD. Each dot represents samples prepared from five different mice. n=5, **p<0.01, ***p<0.001, two-tailed, unpaired t-test. (**c**) Confocal images of the hippocampal CA1 area from 1-month-old $Fus^{\Delta NLS/+}$ and $Fus^{+/+}$ mice. Top: low magnification pictures show the dendritic area of pyramidal cells stained with FUS (green), MAP2 (dendritic marker, magenta), Synapsin 1 (Syn, Synaptic marker, Cyan) and DAPI (Blue). Red box indicates the area imaged in the high magnification images below. n=6 independent $Fus^{+/+}$ and $Fus^{\Delta NLS/+}$ mouse **d**) Higher magnification equivalent to the area highlighted in red in (**c**). (**e**) Volcano plot of the proteomics data showing log2FC of $Fus^{\Delta NLS/+}$ compared to $Fus^{+/+}$ synaptoneurosomes at 6 months. Proteins encoded by mRNA targets of FUS are highlighted in blue. Two-sided t-test with pooled variance. (**f**) Workflow for synaptic marker quantification. Molecular layer of CA1 hippocampal area was imaged by confocal microscopy. Z-stacks were imaged from top (higher Z step with specific signal) to bottom (last step with specific signal) with a Z-step of 0.5 μm. The 40 middle steps were used for quantification. Confocal images were then processed with Huygens professional software for deconvolution. Fiji was used for quantification. Images were first thresholded to only select the specific signal. Images were then binarized and quantification of size and density of synaptic markers was performed using the built-in "Analyze particles", with size exclusion threshold (as described in the Method section). Data were then compiled in open-office and analyzed using Graphpad Prism software. For 1-month-old group, n=6 individual mouse for $Fus^{+/+}$ and $Fus^{\Delta NLS/+}$. For 6-month-old group, n= 5 individual mouse for $Fus^{+/+}$, n = 6 for $Fus^{\Delta NLS/+}$. (**g-h**) Heatmap summarizing the density of the different synaptic markers quantified in the CA1 hippocampal area from $Fus^{\Delta NLS/+}$ 1-month-old (**g**) and 6-month-old (**h**) mice, normalized by the respective control. Mean value of each marker is indicated. Shade of color code for mean variation from 0 (white) to 2 (dark blue). (**i-j**) Heatmap summarizing the cluster area of the different synaptic markers quantified in the CA1 hippocampal area from 1-month-old (**i**) or 6-month-old (**j**) $Fus^{+/+}$ and $Fus^{\Delta NLS/+}$ mice. Mean value of each marker is indicated. Shade of color code for mean variation from 0.01 (white) to 1 (dark red). *p<0.05 **p<0.01, Two-tailed, unpaired t-test. For 1-month-old group, n=6 individual mouse for $Fus^{+/+}$ and $Fus^{\Delta NLS/+}$. For 6-months-old group, n= 5 individual mouse for $Fus^{+/+}$, n = 6 for $Fus^{\Delta NLS/+}$. For Synapsin 1 at 1 month-old, n= 14 fields of view for $Fus^{+/+}$ and 866013 synapses analyzed, 20 fields of view for $Fus^{\Delta NLS/+}$ with 1475455 synapses analyzed. For Synapsin 1 at 6-months-old, n= 19 fields of view for $Fus^{+/+}$

and 1590276 synapses analyzed, 24 fields of view for *Fus*$^{ΔNLS/+}$ with 2138361 synapses analyzed. For vesicular GABA transporter (VGAT) at 1-month-old, n=19 fields of view for *Fus*$^{+/+}$ with 713918 synapses analyzed, 23 fields of view for *Fus*$^{ΔNLS/+}$ with 782790 synapses analyzed. For VGAT at 6-months-old, n=18 fields of view for *Fus*$^{+/+}$ with 553020 synapses analyzed, 24 fields of view for *Fus*$^{ΔNLS/+}$ with 1273325 synapses analyzed. For GABAergic receptors containing α3 subunit ($GABA_Aα3$) at 1-month-old, n=24 fields of view for *Fus*$^{+/+}$ with 1062095 synapses analyzed, 24 fields of view for *Fus*$^{ΔNLS/+}$ with 634958 synapses analyzed. For $GABA_Aα3$ at 6-months-old, n=20 fields of view for *Fus*$^{+/+}$ with 726881 synapses analyzed, 22 fields of view for *Fus*$^{ΔNLS/+}$ with 804732 synapses analyzed. For GluN1 (subunit of NMDAR) at 1-month-old, n=14 fields of view for *Fus*$^{+/+}$ with 704322 synapses analyzed, 20 fields of view for *Fus*$^{ΔNLS/+}$ with 1379868 synapses analyzed. For GluN1 at 6-months-old, n=19 fields of view for *Fus*$^{+/+}$ with 1267271 synapses analyzed, 24 fields of view for *Fus*$^{ΔNLS/+}$ with 1866397 synapses analyzed. For synaptosomal-associated protein 25 (SNAP25) at 1-month-old, n=14 fields of view for *Fus*$^{+/+}$ with 514414 synapses analyzed, 20 fields of view for *Fus*$^{ΔNLS/+}$ with 822138 synapses analyzed. For SNAP25 at 6-months-old, n=19 fields of view for *Fus*$^{+/+}$ with 686248 synapses analyzed, 24 fields of view for *Fus*$^{ΔNLS/+}$ with 1143340 synapses analyzed. For GluA1 (AMPAR subunit) at 1-month-old, n=18 fields of view for *Fus*$^{+/+}$ with 851803 synapses analyzed, 12 fields of view for *Fus*$^{ΔNLS/+}$ with 524156 synapses analyzed. For GluA1 at 6-months-old, n=18 fields of view for *Fus*$^{+/+}$ with 1236519 synapses analyzed, 19 fields of view for *Fus*$^{ΔNLS/+}$ with 1426485 synapses analyzed. For Bassoon at 1-month-old, n=18 fields of view for *Fus*$^{+/+}$ with 393979 synapses analyzed, 12 fields of view for *Fus*$^{ΔNLS/+}$ with 227280 synapses analyzed. For Bassoon at 6-months-old, n=18 fields of view for *Fus*$^{+/+}$ with 1297952 synapses analyzed, 19 fields of view for *Fus*$^{ΔNLS/+}$ with 1229013 synapses analyzed. For pCaMKII at 1-month old, n=19 fields of view for *Fus*$^{+/+}$ with 656996 synapses analyzed, 23 fields of view for *Fus*$^{ΔNLS/+}$ with 814125 synapses analyze. For phospho-calcium/calmodulin-dependent kinase IIα (pCaMKII) at 6-months-old, n=18 fields of view for *Fus*$^{+/+}$ with 345664 synapses analyzed, 24 fields of view for *Fus*$^{ΔNLS/+}$ with 637497 synapses analyzed. For Gephyrin at 1-month-old, n=43 fields of view for *Fus*$^{+/+}$ with 1167770 synapses analyzed, 47 fields of view for *Fus*$^{ΔNLS/+}$ with 1175264 synapses analyzed. For Gephyrin, at 6-months-old, n=38 fields of view for *Fus*$^{+/+}$ with 967407 synapses analyzed, 46 fields of view for *Fus*$^{ΔNLS/+}$ with 1244250 synapses analyzed. For GABAergic receptors containing α1 subunit ($GABA_Aα1$) at 1-month-old, n=24 fields of view for *Fus*$^{+/+}$ with 526148 synapses analyzed, 24 fields of view for *Fus*$^{ΔNLS/+}$ with 417501 synapses analyzed. For $GABA_Aα1$, at 6-months-old, n=20 fields of view for *Fus*$^{+/+}$ with 408930 synapses analyzed, 22 fields of view for *Fus*$^{ΔNLS/+}$ with 498394 synapses analyzed.

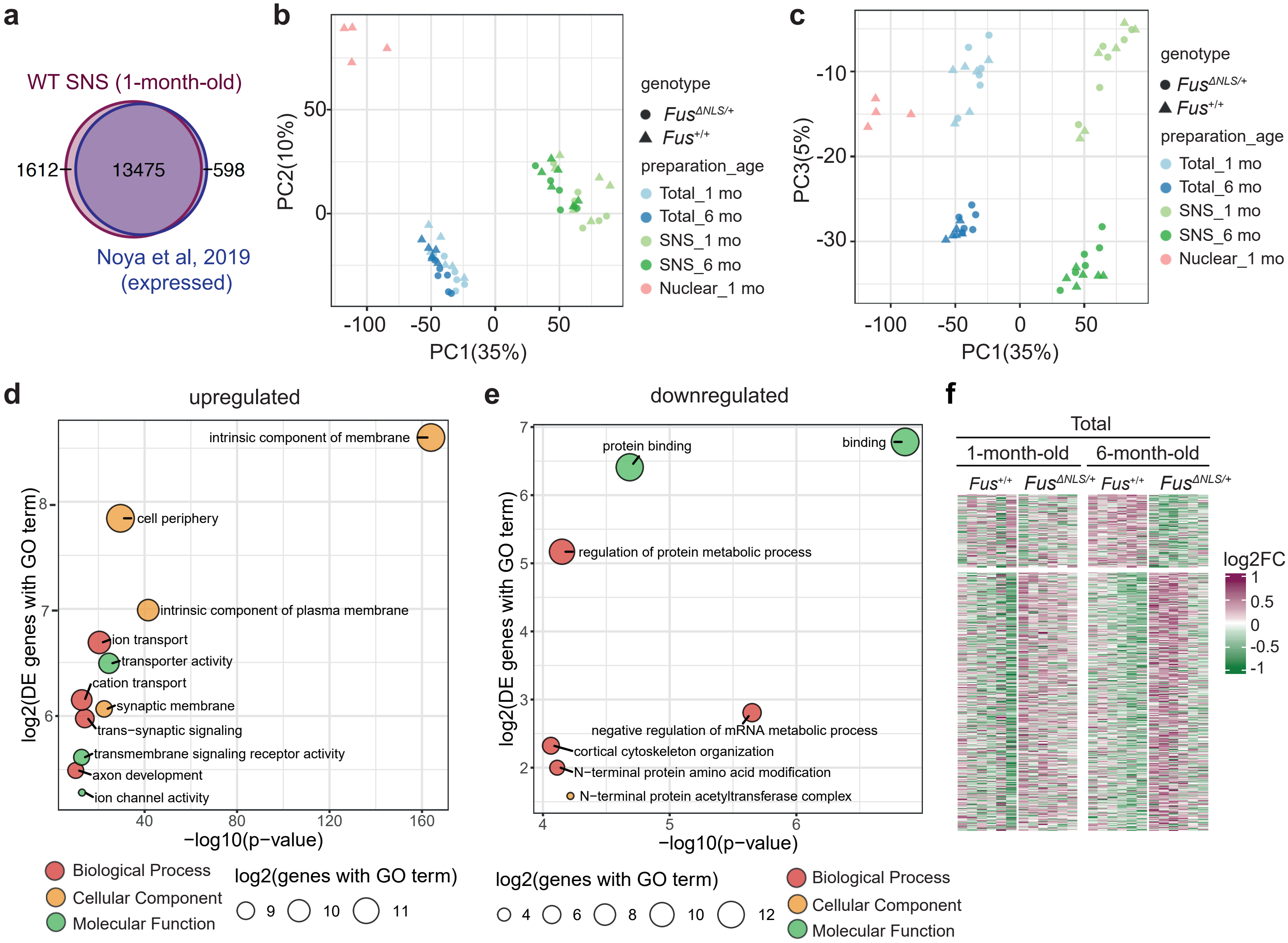

Supplementary Fig. 5 Age-dependent alterations in the synaptic mRNA profile of *Fus*$^{\Delta NLS/+}$ mouse cortex

Supplementary Fig. 5 Age-dependent alterations in the synaptic mRNA profile of *Fus*$^{\Delta NLS/+}$ mouse cortex.

(**a**) Overlap between transcripts expressed in SNS RNA-seq and expressed genes in forebrain synaptic transcriptome reported previously[1]. Expressed genes are all genes with > 10 reads in 2/3 of the replicates (as defined previously[1]). (**b**) Plot of the first and second principal component of all RNA-seq samples and all expressed genes. The genotype is indicated by the symbol and the preparation and age by the color: 1-month-old mice in light and 6-month-old mice in dark colors. (**c**) Plot of the first and third principal component of all RNA-seq samples. (**d**) Selected GO terms enriched among the significantly increased genes at 6 months of age in synaptoneurosomes of *Fus*$^{\Delta NLS/+}$ compared to *Fus*$^{+/+}$ (over representation analysis with all expressed genes in *Fus*$^{+/+}$ SNS at 6 months as background). (**e**) Gene ontology (GO) terms enriched among the significantly decreased RNAs at 6 months of age in synaptoneurosomes of *Fus*$^{\Delta NLS/+}$ compared to *Fus*$^{+/+}$ (over representation analysis with all expressed genes in *Fus*$^{+/+}$ SNS at 6 months as background). P-values in (d-e) are from one-sided hypergeometric tests, p-value unadjusted for multiple comparisons (**f**) Heatmap from the set of up- and downregulated genes between total cortex samples from *Fus*$^{\Delta NLS/+}$ and *Fus*$^{+/+}$ at 1 and 6 months of age. Genes are on the rows and the different total cortex samples on the columns. The color scale indicates the log2FC between the CPM of each sample and mean CPM of the corresponding *Fus*$^{+/+}$ samples at each time point [sample logCPM – mean (logCPM of *Fus*$^{+/+}$ samples)].

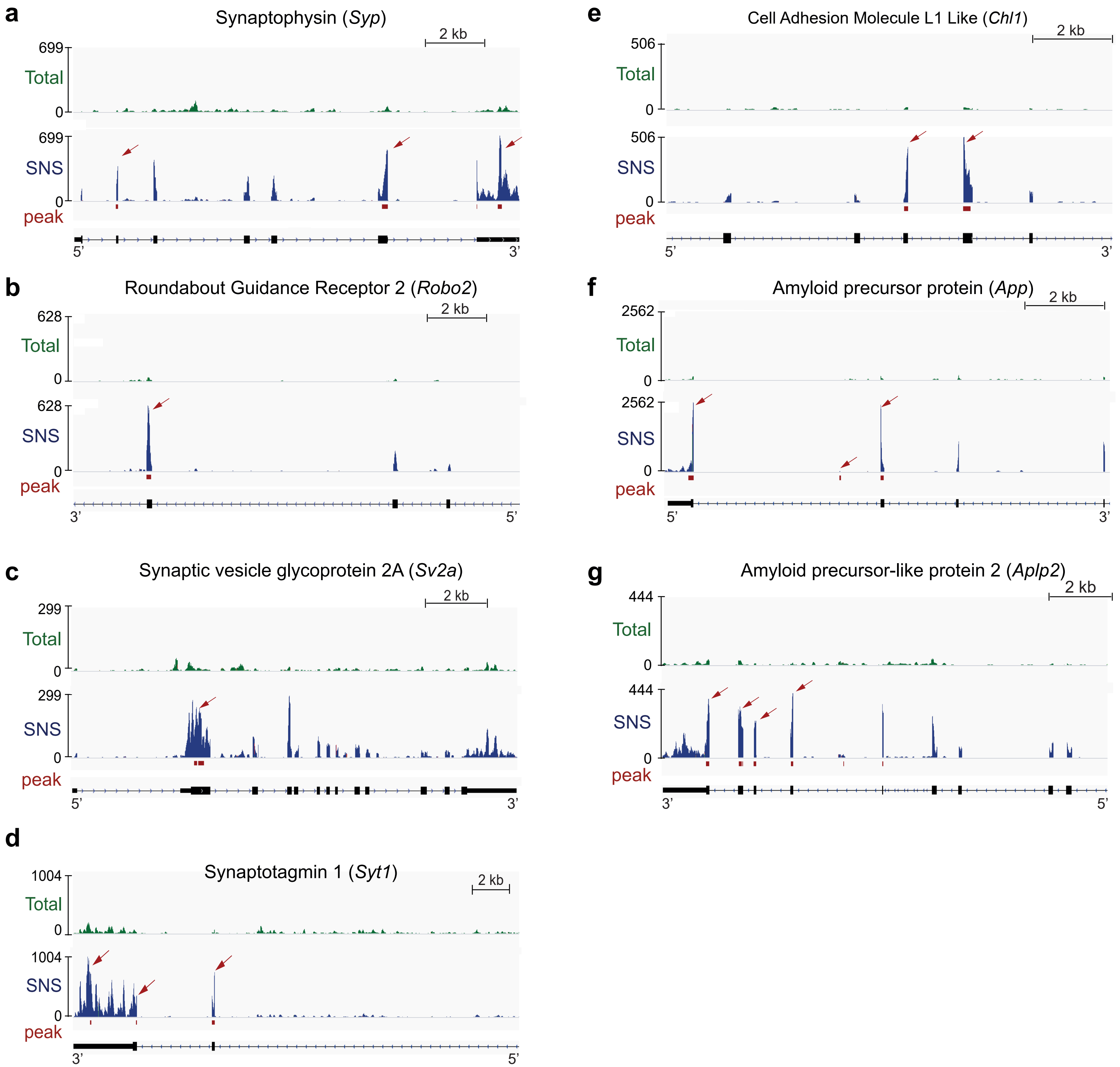

Supplementary Fig. 6 FUS peak locations on postsynaptic FUS RNA targets altered in $Fus^{\Delta NLS/+}$ mice

Supplementary Fig. 6. FUS peak locations on presynaptic and transsynaptic FUS RNA targets altered in *Fus*$^{\Delta NLS/+}$ mice.

CLIP-traces showing FUS binding on (**a**) *Syp* (**b**) *Robo2* (**c**) *Sv2a* (**d**) *Syt1* (**e**) *Chl1* (**f**) *App* (**g**) *Aplp2.*

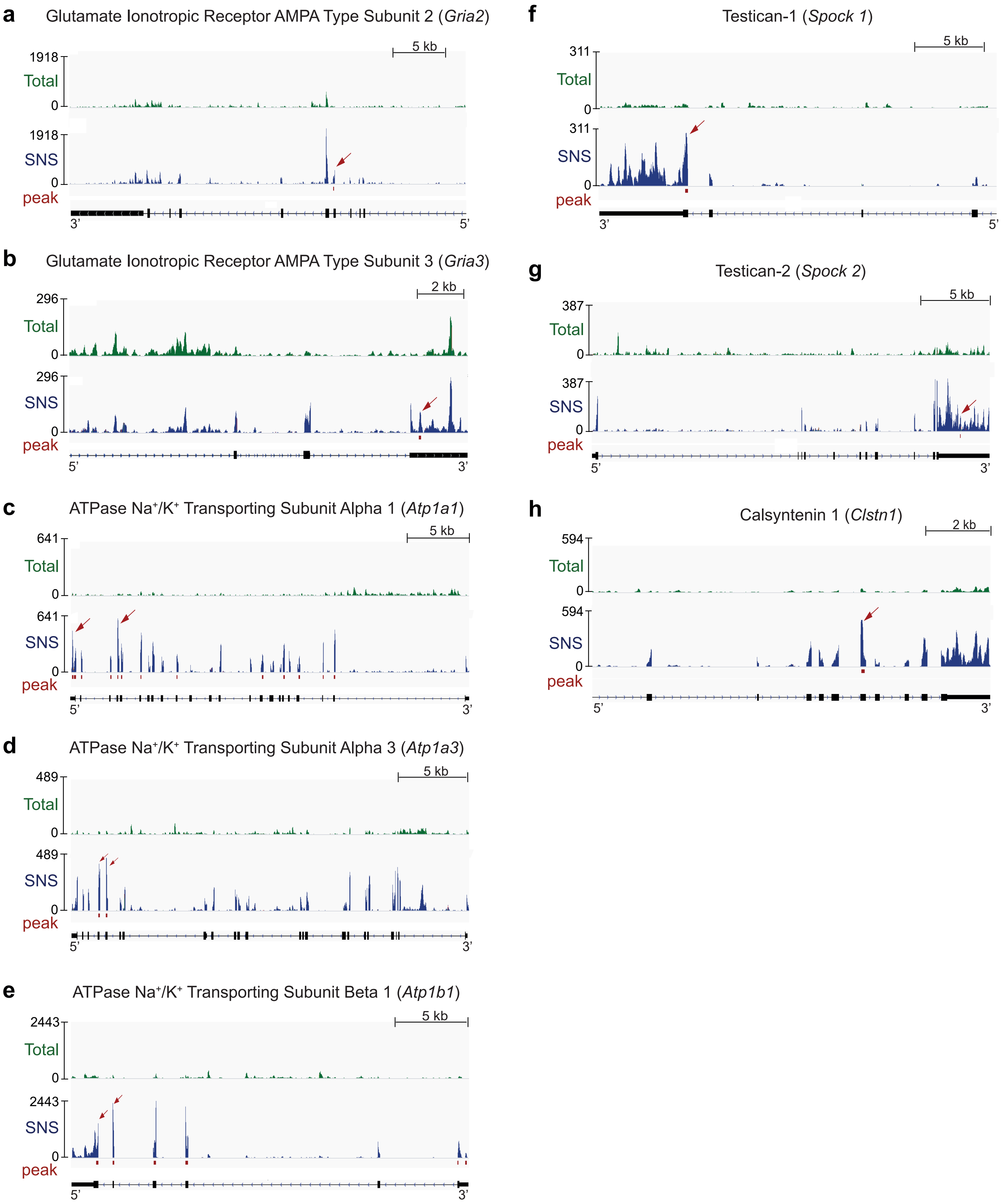

Supplementary Fig. 7 FUS peak locations on postsynaptic FUS RNA targets altered in *Fus*$^{\Delta NLS/+}$ mice

Supplementary Figure 7. FUS peak locations on postsynaptic FUS RNA targets altered in *Fus*$^{\Delta NLS/+}$ mice.

CLIP-traces showing FUS binding on (**a**) *Gria2* (**b**) *Gria3* (**c**) *Atp1a1* (**d**) *Atp1a3* (**e**) *Atp1b1* (**f**) *Spock1* (**g**) *Spock2* (**h**) *Clstn1***.**

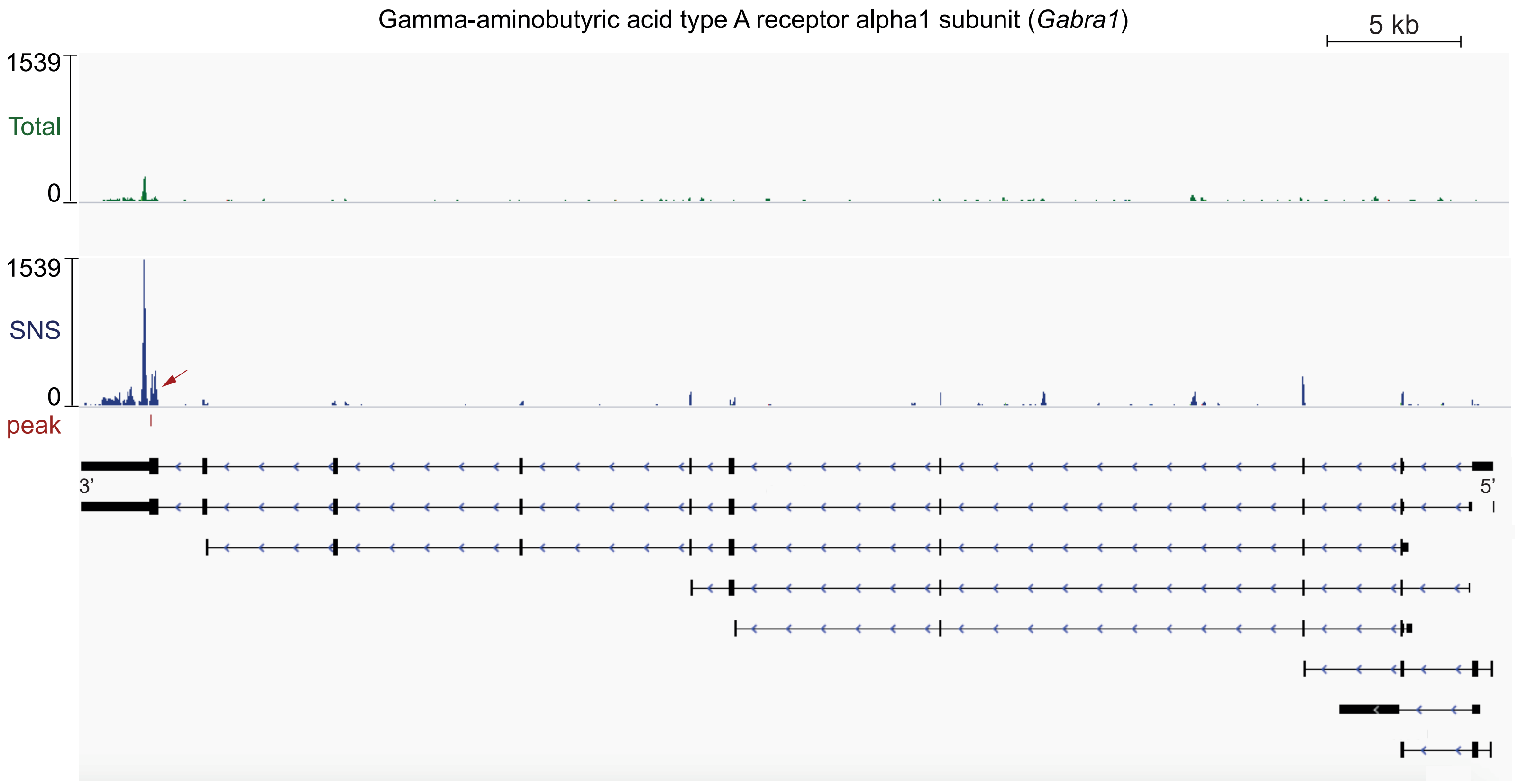

Supplementary Fig. 8 FUS binding on *Gabra1* RNA

Supplementary Figure 8. FUS binding on *Gabra1* RNA.

CLIP-traces showing FUS binding to the long 3'UTR containing isoform of *Gabra1.*

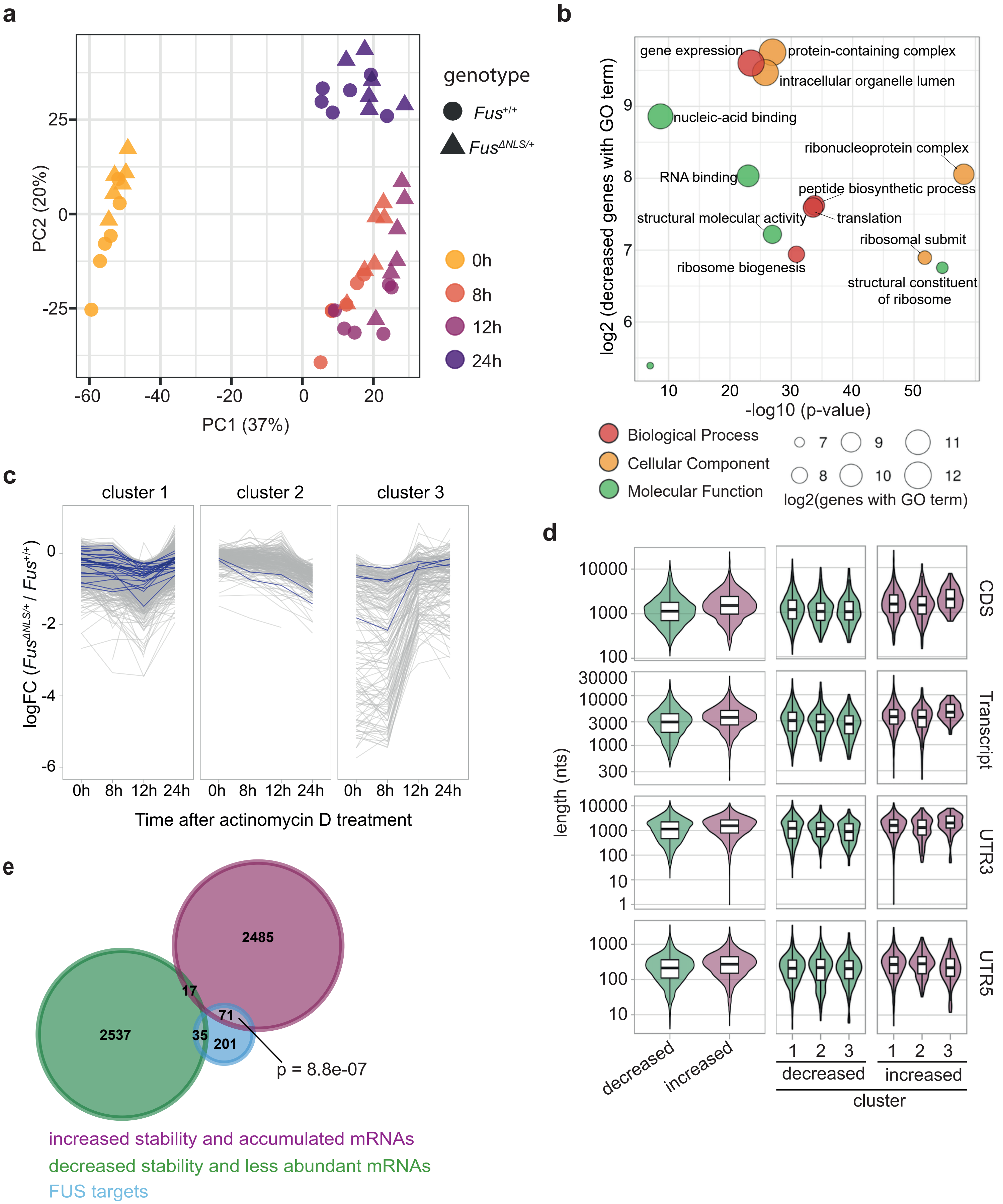

Supplementary Fig. 9 Cytoplasmic FUS accumulation in *Fus*$^{ΔNLS/+}$ neurons leads to altered mRNA stability

Supplementary Figure 9: Cytoplasmic FUS accumulation in *Fus*$^{\Delta NLS/+}$ neurons leads to altered mRNA stability.

(**a**) First and second principal component of all samples in the stability assay, percentage of variance explained in brackets. The color indicates the time point and the symbol the genotype of each sample. (**b**) Selected GO terms enriched in the set of significantly decreased RNAs at 12h. One-sided hypergeometric test, p-value unadjusted for multiple comparisons. (**c**) Hierarchical clustering of the log2 fold changes of the set of decreased RNAs to three groups. The x-axis shows the time points and the y-axis the logFC (*Fus*$^{\Delta NLS/+}$/*Fus*$^{+/+}$); synaptic FUS targets are highlighted in blue. (**d**) Violin and box plot with the length distribution of the longest transcript, CDS, 3'UTR and 5'UTR of all significantly decreased and increased mRNAs at any time point (left column). Decreased RNAs are shorter than increased mRNAs (one-sided t-test: t = -10.109, df = 4732.2, p-value < 2.2e-16 (Transcript), t = -9.8064, df = 4369.3, p-value < 2.2e-16 (CDS), t = -6.8906, df = 4605.9, p-value = 3.2e-12 (UTR3), t = -7.1702, df = 4542.2, p-value = 4.4e-13 (UTR5), n = 2506 decreased and n = 2266 increased mRNAs). The distributions are split into the three clusters of the decreased mRNAs (middle column, cluster 1 n = 2033, cluster 2 n = 268, cluster 3 n = 205 mRNAs) and the increased mRNAs (right column, cluster 1 n = 2046, cluster 2 n = 155, cluster 3 n = 65 mRNAs). The box ranges from the first to the third quartile and includes the median. Box plots indicate median (middle line), 25th, 75th percentile (box) and the lower/upper whiskers extend to the minimal/maximal value no further than 1.5 × inter-quartile range from the hinge. (**e**) Euler diagram with three sets: the synaptic FUS targets (blue); the RNAs with increased stability at any time point or accumulation in SNS of 6-month-old *Fus*$^{\Delta NLS/+}$ mice (magenta); the RNAs with decreased stability at any time point or reduced abundance in SNS of 6-month-old *Fus*$^{\Delta NLS/+}$ mice (green). FUS targets are enriched in the set of genes with increased stability and accumulation (p-value = 8.8e-07, one-sided Fisher's exact test), but not in the set of genes that are less abundant or less stable (p = 1, one-sided Fisher's exact test).

Supplementary references

1. Noya, S. B. *et al.* The forebrain synaptic transcriptome is organized by clocks but its proteome is driven by sleep. *Science (80-.).* **366**, (2019).
