# Supplementary material for: Synaptic FUS accumulation triggers early misregulation of synaptic RNAs in a mouse model of ALS
Source: Nat Commun. 2021 May 21;12:3027. doi: 10.1038/s41467-021-23188-8 (PMC8140117; doi:10.1038/s41467-021-23188-8)
Supplement: Supplementary file 3 — Description of Additional Supplementary Files [file 41467_2021_23188_MOESM3_ESM.pdf]

Description of Additional Supplementary Files

Supplementary data 1. Lists of synaptic FUS RNA-targets
The file reports the peak locations and number of clusters of FUS synaptic RNA targets identified from FUS CLIP-seq using synaptoneurosomes. For comparison, it also includes the results from the differential gene expression analysis in synaptoneurosomes ($Fus^{\Delta NLS/+}$ vs $Fus^{+/+}$) at 6 months of age and stability assay results for these FUS synaptic targets.

Supplementary data 2. Statistical analysis of synaptic density
The table reports statistical analysis of density of the synaptic markers analyzed from a minimum of 2 images from at least 4 animals per genotype ($Fus^{+/+}$ and $Fus^{\Delta NLS/+}$) at 1 and 6 months of age. Two-tailed, Unpaired t-test. Unpaired t-test statistics, p-values, specific t-distribution (t), degrees of freedom (DF) and sample size are listed.

Supplementary data 3. Statistical analysis of synaptic cluster area
The file reports statistical analysis of area of the synaptic markers analyzed from a minimum of 2 images from at least 4 animals per genotype ($Fus^{+/+}$ and $Fus^{\Delta NLS/+}$) at 1 and 6 months of age. Two-tailed, Unpaired t-test. Unpaired t-test statistics, p-values, specific t-distribution (t), degrees of freedom (DF) and sample size are listed.

Supplementary data 4. Differential gene expression analysis $Fus^{+/+}$ vs $Fus^{\Delta NLS/+}$ total cortex (1-month-old)
The file contains the results from the differential gene expression analysis with edgeR (quasi-likelihood F-test) comparing the total cortex from $Fus^{\Delta NLS/+}$ versus $Fus^{+/+}$ at 1 month of age. Rows are the tested gene, columns include the genomic coordinates, the Ensembl gene ID, name and biotype, the logFC and logCPM of the comparison, as well as the F statistic, raw p-value, FDR (false-discovery rate) and minus log10 (p-value).

Supplementary data 5. Differential gene expression analysis $Fus^{+/+}$ vs $Fus^{\Delta NLS/+}$ total cortex (6-months-old)
The file contains the results from the differential gene expression analysis with edgeR (quasi-likelihood F-test) comparing the total cortex from $Fus^{\Delta NLS/+}$ versus $Fus^{+/+}$ at 6 months of age. Rows are the tested gene, columns include the genomic coordinates, the Ensembl gene ID, name and biotype, the logFC and logCPM of the comparison, as well as the F statistic, raw p-value, FDR (false-discovery rate) and minus log10(p-value).

Supplementary data 6. Differential gene expression analysis $Fus^{+/+}$ vs $Fus^{\Delta NLS/+}$ synaptoneurosomes (1-month-old)
The file contains the results from the differential gene expression analysis with edgeR (quasi-likelihood F-test) comparing the synaptoneurosomes from $Fus^{\Delta NLS/+}$ versus $Fus^{+/+}$ at 1 month of age. Rows are the tested gene, columns include the genomic coordinates, the Ensembl gene ID, name and biotype, the logFC and logCPM of the comparison, as well as the F statistic, raw p-value, FDR (false-discovery rate) and minus log10(p-value).

Supplementary data 7. **Differential gene expression analysis *Fus*$^{+/+}$ vs *Fus*$^{\Delta NLS/+}$ synaptoneurosomes (6-months-old)**
The file contains the results from the differential gene expression analysis with edgeR (quasi-likelihood F-test) comparing the synaptoneurosomes from *Fus*$^{\Delta NLS/+}$ versus *Fus*$^{+/+}$ at 6 months of age. Rows are the tested gene, columns include the genomic coordinates, the Ensembl gene ID, name and biotype, the logFC and logCPM of the comparison, as well as the F statistic, raw p-value, FDR (false-discovery rate) and minus log10(p-value).

Supplementary data 8. **Differential gene expression analysis of stability experiment**
The file contains the results from the differential genes expression analysis with edgeR (quasi-likelihood F-test) comparing *Fus*$^{\Delta NLS/+}$ versus *Fus*$^{+/+}$ at 0h, 8h, 12h and 24h post actinomycin D treatment. Rows are the tested gene, columns include the genomic coordinates, the Ensembl gene ID, name and biotype, the logFC and logCPM of the comparison, as well as the F statistic, raw p-value, FDR (false-discovery rate) and minus log10(p-value).

Supplementary data 9. **List of antibodies**
The file contains the list of antibodies and dilutions used for the experiments

Supplementary data 10. **Lists of qRT-PCR primers**
The file contains the primer sequences used for performing qRT-PCR.
