# Supplementary material for: Synaptic FUS accumulation triggers early misregulation of synaptic RNAs in a mouse model of ALS
Source: Nat Commun. 2021 May 21;12:3027. doi: 10.1038/s41467-021-23188-8 (PMC8140117; doi:10.1038/s41467-021-23188-8)
Supplement: Supplementary file 14 — Reporting summary [file 41467_2021_23188_MOESM14_ESM.pdf]

nature research

Corresponding author(s): Polymenidou
Last updated by author(s): Mar 16, 2021

Reporting Summary

Nature Research wishes to improve the reproducibility of the work that we publish. This form provides structure for consistency and transparency in reporting. For further information on Nature Research policies, see our Editorial Policies and the Editorial Policy Checklist.

Statistics

For all statistical analyses, confirm that the following items are present in the figure legend, table legend, main text, or Methods section.

n/a	Confirmed	
☐	☒	The exact sample size (n) for each experimental group/condition, given as a discrete number and unit of measurement
☐	☒	A statement on whether measurements were taken from distinct samples or whether the same sample was measured repeatedly
☐	☒	The statistical test(s) used AND whether they are one- or two-sided<br>*Only common tests should be described solely by name; describe more complex techniques in the Methods section.*
☐	☒	A description of all covariates tested
☐	☒	A description of any assumptions or corrections, such as tests of normality and adjustment for multiple comparisons
☐	☒	A full description of the statistical parameters including central tendency (e.g. means) or other basic estimates (e.g. regression coefficient) AND variation (e.g. standard deviation) or associated estimates of uncertainty (e.g. confidence intervals)
☐	☒	For null hypothesis testing, the test statistic (e.g. F, t, r) with confidence intervals, effect sizes, degrees of freedom and P value noted<br>*Give P values as exact values whenever suitable.*
☒	☐	For Bayesian analysis, information on the choice of priors and Markov chain Monte Carlo settings
☒	☐	For hierarchical and complex designs, identification of the appropriate level for tests and full reporting of outcomes
☐	☒	Estimates of effect sizes (e.g. Cohen's d, Pearson's r), indicating how they were calculated

Our web collection on statistics for biologists contains articles on many of the points above.

Software and code

Policy information about availability of computer code

Data collection	Leica X suites for microscopy
Data analysis	Leica LAS X suites, Imaris 9.5.1 from Oxford Instrument, Fiji Image J 1.52p, Huygens Professional software (19.10.0p0) were used for microscopy image analysis. Fiji version 2.0.0-rc-69/1.52p was used for quantification of western blots.<br>Sequencing data was processed with ARMOR (https://github.com/csoneson/ARMOR) using TrimGalore! (0.5.0), STAR (2.4.2a), picard tools (2.18.4), bedtools (v2.27.1), Salmon (0.10.2), R (3.6.0) and the tximeta and edgeR packages. Details and the used parameters are stated in the Methods section. Over representation analysis was performed with the limma R package. CLIP-seq peaks were analysed with CLIPper, HOMER and R (3.6.0). All code is deposited on github: CLIP-seq analysis (https://github.com/khembach/FUS_CLIPseq), RNA-seq analyis (https://github.com/khembach/FUS_RNAseq) and figures (https://github.com/khembach/FUS_paper), and stability assay analysis (https://github.com/khembach/FUS_stability).<br>Proteomics analysis was performed with MaxQuant (version 1.6.2.3) and SRMSevice (http://github.com/protViz/SRMService).

For manuscripts utilizing custom algorithms or software that are central to the research but not yet described in published literature, software must be made available to editors and reviewers. We strongly encourage code deposition in a community repository (e.g. GitHub). See the Nature Research guidelines for submitting code & software for further information.

Data

Policy information about availability of data

All manuscripts must include a data availability statement. This statement should provide the following information, where applicable:

- Accession codes, unique identifiers, or web links for publicly available datasets
- A list of figures that have associated raw data
- A description of any restrictions on data availability

Raw data were deposited at ArrayExpress Archive of Functional Genomics Data (https://www.ebi.ac.uk/arrayexpress/). Accession codes for RNA-seq are E-MTAB-9212 (total cortex and SNS) and E-MTAB-10104 (stability experiment); E-MTAB-9211 for CLIP-seq libraries. The mass spectrometry proteomics data have been deposited to the ProteomeXchange Consortium via the PRIDE partner repository with the dataset identifier PXD024075. Full western blots and quantification data are included in the Source Data file.

Field-specific reporting

Please select the one below that is the best fit for your research. If you are not sure, read the appropriate sections before making your selection.

☒ Life sciences ☐ Behavioural & social sciences ☐ Ecological, evolutionary & environmental sciences

For a reference copy of the document with all sections, see nature.com/documents/nr-reporting-summary-flat.pdf

Life sciences study design

All studies must disclose on these points even when the disclosure is negative.

Sample size	For CLIP experiment from synaptoneurosomes we use material from 200 mice. For this experiment we did not use replicates (reasons - requirement of huge number of mice). We did not use any stastical method to predetermine the sample size.
Data exclusions	No data were excluded from the analysis
Replication	We used replicates and the specific number of replicates that we used for each experiments are mentioned in the paper.
Randomization	Mice were randomly assigned to synaptoneurosome or total cortex samples. All quantification have been performed in a non biased way
Blinding	Investigator who analysed the imaging data were blinded. Investigators of CLIP-seq, RNA-seq and proteomics data collection and analyses were not blinded, because mice were genotyped and assigned to groups accordingly and knowledge of group allocation was required for statistical analyses and figure preparation.

Reporting for specific materials, systems and methods

We require information from authors about some types of materials, experimental systems and methods used in many studies. Here, indicate whether each material, system or method listed is relevant to your study. If you are not sure if a list item applies to your research, read the appropriate section before selecting a response.

Materials & experimental systems

n/a	Involved in the study	
☐	☒	Antibodies
☒	☐	Eukaryotic cell lines
☒	☐	Palaeontology and archaeology
☐	☒	Animals and other organisms
☒	☐	Human research participants
☒	☐	Clinical data
☒	☐	Dual use research of concern

Methods

n/a	Involved in the study	
☒	☐	ChIP-seq
☒	☐	Flow cytometry
☒	☐	MRI-based neuroimaging

Antibodies

Antibodies used	FUS (Bethyl Laboratories; Catalog No: A300-293A), FUS (Bethyl Laboratories; Catalog No: A300-294A), FUS (Santa Cruz Biotechnology, clone 4H11, Catalog No. sc-47711,), PSD-95 (invitrogen, MA1-045), Spinophilin (Synaptic systems, Catalog No. 399 003), Synapsin 1 (Synaptic systems, Catalog No. 106 011BT), Bassoon (Synaptic systems, Catalog No. 141 004), GluR1 (Sigma Aldrich, Catalog No. 04-855, clone C3T), P-CAMKIIa (Cell signaling, Catalog No. 12716,D21E4), anti-Neurofilament H (Biolegend, Catalog no. SMI 31P, clone SMI 31), NMDAR1(Biolegend, Catalog No. MMS-5145, Clone N308/48), NMDAR2B (Invitrogen, Catalog No. 71-8600), GRP78 BiP (abcam, Catalog No. ab21685), MAP2 (sigma Aldrich, Catalog No. M1406), SYP (Santa Cruz Biotechnology, sc-9116),GABA-A receptor α 1(Synaptic systems, Catalog No. 224 204), GABA-A receptor α3 (Synaptic systems, Catalog No. 224 303), Gephyrin (Synaptic systems, Catalog No. 147 011), VGAT (Synaptic systems, Catalog No. 131 005), β-Actin (sigma Aldrich, Catalog No. A5441),

	SNAP 25 (Synaptic systems, Catalog No. 111 004).
Validation	All antibodies were validated by the manufacturing company. We followed the antibody dilutions according to the manufacturer's website.

Animals and other organisms

Policy information about studies involving animals; ARRIVE guidelines recommended for reporting animal research

Laboratory animals	We used adult C57/Bl6 mice and Fus+/+/FusΔNLS/+ mice with genetic background (C57/BL6), of both sex at 1 and 6 months of age. We also prepared primary cultures from P0 pups from these mouse strains.
Wild animals	Study did not involve wild animals
Field-collected samples	Study did not involve samples collected from the field
Ethics oversight	Mice housing and breeding were in accordance with the Swiss Animal Welfare Law and in compliance with the regulations of the Cantonal Veterinary Office, Zurich

Note that full information on the approval of the study protocol must also be provided in the manuscript.
